# Purposes of Smartphone Use in Later Life: User Perspectives on Digital Daily Life in Germany

**DOI:** 10.1093/geroni/igaa057.2993

**Published:** 2020-12-16

**Authors:** Friedrich Wolf, Frank Oswald

**Affiliations:** 1 Interdisciplinary Ageing Research (IAW), Frankfurt, Hessen, Germany; 2 Interdisciplinary Ageing Research, Frankfurt, Hessen, Germany

## Abstract

Smartphones affect everyday life of people across the lifespan with increasing relevance in later life. Although half of older adults in Germany (65+) use smartphones and the numbers of users are growing, only few studies address the integration of older adult’s smartphone use in everyday life as well as its potential effects on social context and wellbeing, which is been done in this study. Data are drawn from surveying the devices of 35 older adults (age 61-77 years; 60% women), allowing to objectively monitor real time smartphone use over the course of a regular week. In addition a semi-standardized ambulatory assessment procedure is administered four times a day to capture the everyday situation of smartphone use as well as aspects of user experience, affect and social context. Preliminary findings show inter- and intra-individual differences in everyday smartphone use in relation to individual differences in social context and wellbeing.

